# Sialoadhesin deficiency does not influence the severity of lupus nephritis in New Zealand Black x New Zealand White F1 mice

**DOI:** 10.1186/ar4364

**Published:** 2013-11-01

**Authors:** Dana Kidder, Hannah E Richards, Paul A Lyons, Paul R Crocker

**Affiliations:** 1 Division of Cell Signalling and Immunology, College of Life Sciences, University of Dundee, Dow Street, Dundee DD1 5EH, United Kingdom; 2 Cambridge Institute for Medical Research and Department of Medicine, University of Cambridge, Hills Road, Cambridge CB2 0XY, United Kingdom

## Abstract

**Introduction:**

Systemic lupus erythematosus (SLE) is a chronic inflammatory condition with multisystem involvement. One of the key features of the disease is the upregulation of type I interferons, resulting in the so-called “interferon signature”. Recent flow cytometric and transcriptomic studies identified Sialoadhesin (Sn, CD169) as an important interferon-induced blood monocyte biomarker in diseased patients. To investigate a potential causative role of Sn in SLE, we generated NZBWF1 (New Zealand Black x New Zealand White F1) mice lacking Sn and compared onset and progression of disease with NZBWF1 expressing normal levels of Sn.

**Methods:**

Sn expression in renal tissues of pre-diseased and diseased NZBWF1 mice was evaluated by Quantitative real time PCR (QPCR) and immunohistochemistry. Sn^−/−^ NZBWF1 mice were generated by speed congenics. Disease severity of Sn^+/+^ and Sn^−/−^ NZBWF1 mice was assessed by serum immunoassays, flow cytometry, light microscopy and immunohistochemistry.

**Results:**

Renal tissues from proteinuric NZBWF1 mice exhibited a significant upregulation of Sn mRNA and protein expression following disease onset. Further immunohistochemical analysis showed that Sn^+^ macrophages assumed a distinct periglomerular distribution and, unlike CD68^+^ macrophages, were not present within the glomeruli. Analysis of disease severity in Sn^*−/−*^ and Sn^*+/+*^ NZBWF1 mice revealed no significant differences in the disease progression between the two groups although Sn-deficient mice showed a more rapid onset of proteinuria.

**Conclusions:**

These data confirm a positive correlation of Sn with disease activity. However, Sn deficiency does not have a significant effect on the severity and progression of lupus nephritis in the NZBWF1 mouse model.

## Introduction

Systemic lupus erythematosus (SLE) is an autoimmune chronic inflammatory condition with a multi-system pattern of involvement. SLE is the prototype of systemic autoimmunity of unknown aetiology [1]. The disease is characterized by loss of self-tolerance and accelerated apoptosis with subsequent release of nucleosomal material, which is a major target for immune responses [2]. Failure to clear these apoptotic materials results in expansion of autoreactive lymphocytes, with increased formation of autoantibody [3,4]. Increased levels of antigen-antibody immune-complexes lead to deposition of these in various tissues, causing inflammation and damage by activation of other immune pathways, for example, the complement system. The kidneys are prime sites for immune complex deposition, resulting in glomerulonephritis [5]. Type I IFNs, particularly IFNα, were found to be markedly upregulated in patients with active SLE [6]. Interestingly, there was a tendency in the high-IFN group to develop renal, haematological and central nervous system (CNS) disease. These findings confirmed a correlation between this interferon signature and disease severity.

Sialoadhesin, (Sn, CD169, siglec-1) is the prototype of sialic acid-binding immunoglobulin-like lectins (Siglecs) [7]. Sn is a macrophage-restricted adhesion molecule that is expressed on macrophages of the marginal metalophilic zone and subcapsular sinus in spleen and lymph nodes respectively. Sn expression can be induced on other macrophages under inflammatory conditions, especially under the influence of IFNα [8-11]. Consistent with this observation, Sn was identified as a biomarker for the type I IFN-signature in SLE [12,13]. The expression of Sn on blood monocytes was found to correlate with disease severity and other established biomarkers of the disease. However, the role of Sn in this complex polygenic disease is not clear. The correlation between Sn expression and disease severity suggested a pro-inflammatory role for the molecule. In support of this hypothesis, Sn deficiency was found to ameliorate disease severity in other mouse models of autoimmunity [14-17]. Alternatively, Sn expression might reflect disease activity without a direct role in the disease pathogenesis. The evidence for either argument in SLE is lacking.

These findings prompted us to examine the potential role of Sn in a well-established murine model of SLE, New Zealand black X New Zealand white F1 (NZBWF1) mice. In this study we examined the expression of Sn in renal cortical tissues prior to disease onset and in diseased mice. We found that the frequency of Sn^+^ macrophages directly correlated with histological severity. To determine the impact of Sn in disease pathogenesis, we generated Sn-deficient (Sn^*−/−*^) NZBWF1 mice using a speed congenics approach. Surprisingly, there was no significant difference in disease severity between wild-type (WT) and Sn-deficient mice. These findings indicate that Sn represents a biomarker for the disease rather than contributing a dominant role in the pathogenesis.

## Methods

### Mice

New Zealand white (NZW), New Zealand black (NZB) and NZBWF1 mice were purchased from Harlan Laboratories (Oxford, UK) or bred and maintained by Biological Services at the University of Dundee, (Dundee, UK). The generation of Sn-deficient mice was previously reported by our laboratory [18]. The Sn-deficient mice that were initially used to generate Sn^−/−^ NZBWF1 mice were intercross offspring of heterozygotes backcrossed for more than 20 generations onto a C57BL/6 background. Mice were housed under specific-pathogen-free conditions. The animal protocols used in this study were approved by the Ethical Review Committee of the University of Dundee. All procedures involving living animals were conducted according to the requirement of the United Kingdom Home Office Animals Scientific procedures Act (1986), under PPL60/3856.

### Generation of Sn^−/−^NZBWF1 mice

The generation of NZBWF1 mice deficient for Sn was done in three phases: (1) generation of parallel NZB and NZW mice that are heterozygous for Sn; (2) crossing Sn heterozygous mice for each strain to generate NZB and NZW mice wild-type and deficient for Sn; (3) intercrossing WT or Sn-deficient male NZW mice with WT or Sn-deficient NZB mice, to generate Sn WT and Sn^−/−^ NZBWF1 mice.

Speed congenics [19] was employed to transfer a set of predefined NZB and NZW susceptibility loci (Additional file 1) to recipient mice that are heterozygous for Sn. Monitoring the introgression of these loci was examined by genotyping with microsatellite markers (20 for NZB and 25 for NZW).

Ear biopsies from mice in each progeny were genotyped initially for the presence of the Sn mutation. All Sn heterozygous NZB and NZW male mice were then screened for relevant polymorphic markers (Additional file 1). Once all required susceptibility loci were introgressed, further genotyping was done for Sn only. All PCRs were done using the GoTaq®Flexi DNA polymerase PCR kit (Promega, Southamptom, UK) using 100 mM 2’-deoxynucleoside 5’-triphosphate (dNTP) purchased from Invitrogen. The primer sets used in genotyping of NZW polymorphic markers were designed using Primer Express software 2.0 (Applied Biosystems, California, USA), also found in Additional file 1.

### Quantitative reverse transcription PCR (qRT-PCR)

RNA extraction was done using RNeasy mini kit (Qiagen, Manchester, UK) according to manufacturer’s instructions. In this protocol, renal cortical samples from the left kidney were lysed in the presence of denaturing guanidine-thiocyanate-containing buffer to inactivate RNases. Sample homogenization was done with a rotor-stator homogenizer using homogenization tubes containing 1.4-mm ceramic beads (Precelys, Peqlab, Southampton, UK).

The concentration of the extracted RNA was measured based on the absorbance at 260 nm (A260) in a NanoDrop 1000 spectrophotometer (Thermoscientific, Massachusetts,Boston, USA). The purity of RNA was estimated by measuring the ratio of the readings at 260 nm and 280 nm (A260/A280). Sample ratios between 1.9 and 2.1 were regarded as acceptable for cDNA preparation.

cDNA synthesis was performed using a QuantiTect reverse transcription kit (Qiagen). All incubations were done in a thermocycler (Bio-Rad, Hertfordshire, UK) for the specified temperatures and durations. All cDNA samples were stored at −20°C until used in qRT-PCR experiments. cDNA was subsequently quantified with a StepOne Plus detection system (Applied Biosystems, California, USA) using specific primers for each marker and a SYBR Green-based detection system (Applied Biosystems, California, USA). Input RNA was normalised between samples using GAPDH as an endogenous control. Fold upregulation of the target gene was calculated as ratio target gene expression (experimental/control). Primers used in these experiments were as follows: SnD2 5’-CCCAGCCCCCCCACTAT-3’ and 5’-AAGTTCCTCTCCATGCCTTCAC-3’; CD68 5’-CTTCCCACAGGCAGCACAG-3’ and 5’-AATGATGAGAGGCAGCAAGAGG-3’; IFNαR1 5’-TGGTGGTTCTGTCTCGGTGTT-3’ and 5’-GCTACGGCAGGATTAAAAATCG-3’; IFNγ 5’-AGCTCTTCCTCATGGCTGTT-3’ and 5’-TTTGCCAGTTCCTCCAGATA-3’; IL-1β 5’-AAGTGATATTCTCCATGAGCTTTG-3’ and 5’-TTCTTCTTTGGGTATTGCTTGG-3’.; TNFα 5’-CGTCGTAGCAAACCACCAAG-3’ and 5’-TTGAAGAGAACCTGGGAGTAGACA-3’; IL-6 5’-TGGGAAATCGTGGAAATGAG-3’ and 5’-CTCTGAAGGACTCTGGCTTTG-3’.

### Measurement of proteinuria

Mice were monitored for disease onset by testing urine with a test strip (Uristix, Bayer, Berlin, Germany) for protein excretion on a weekly basis until proteinuria was detected and then thrice weekly thereafter. Results were graded according to the extent of proteinuria as follows: 0 = negative or trace, + = 30 mg/dl, ++ = 100 mg/dl, +++ = 300 mg/dl and ++++ = >2,000 mg/dl. Onset of disease was defined as two consecutive readings equal to or more than ++ (100 mg/dl).

### Analysis of serum anti-ss/dsDNA

Sera from BWF1 mice were analysed for IgM and IgG anti-ss/dsDNA titres using ELISA. Then, 96-well microtitre plates were coated with 20 μg/ml of poly-L lysine (Sigma, Dorset, UK) in deionised water overnight at 4°C. Plates were washed three times with PBS and 20 μg/ml calf thymus DNA was added for 3 hours at 37°C. Plates were washed three times with PBS + 0.05% Tween (PBST) and non-specific binding was blocked using PBS + 3% BSA for 1 hour at 37°C. Following three washes with PBST, serial dilutions of sera were added in triplicate for 2 hours at 37°C. Sera dilutions were started at 1/100 to 1/6,400 in PBS + 0.5% BSA. Plates were washed three times with PBS + 0.5% BSA. Secondary antibodies diluted in PBS + 0.5% BSA used for detection of immunoglobulin (Ig)M and IgG anti-ss/dsDNA included alkaline phosphatase-conjugated goat anti-mouse IgM (Sigma, Dorset, UK) or horseradish peroxidase (HRP)-conjugated goat anti-mouse IgG (Sigma, Dorset, UK). This was followed by five washes with PBST. Plates were developed either with p-nitrophenyl phosphate substrate (Sigma, Dorset, UK) for alkaline phsophatase-conjugated secondary antibodies or TMB substrate (BD Bioscience, Oxford, UK) for HRP-conjugated secondary antibodies. Plate readings were analysed at 405 nm for the former and 450 nm for the latter. Pooled sera from old female NZBWF1 mice were used as a positive control in all experiments.

### Flow cytometry

All staining and washes were carried out in ice-cold fluorescence-activated cell sorting (FACS) buffer (PBS + 0.5% BSA + 2 mM ethylenediaminetetraacetic acid (EDTA) + 0.05% w/v sodium azide) and tubes incubated on ice to minimise antibody internalisation. Non-specific binding was limited by incubation with 0.25 μg of anti-CD16/CD32 (Fc blocking antibody 2.4G2, eBioscience, San Diego, California, USA) per 1 million cells in 25 μl for 15 minutes. Surface labelling was carried out for 30 minutes with optimal concentrations of the relevant antibodies as decided by prior antibody titrations. The antibodies used in this study include rat anti-mouse; -CD4 PerCp-Cyanine5.5 (clone RM4-5, eBioscience, San Diego, California, USA); -CD8a BD Horizon V500 (clone 53–6.7, BD eBioscience, San Diego, California, USA); -CD19 FITC (clone eBio1D3, eBioscience, San Diego, California, USA); -B220 Alexa Fluor 700 (clone RA3-6B2, eBioscience, San Diego, California, USA), and -CD69 BD Horizon V450 (clone H1.2 F3, BD Biosciences). Cells were washed twice with FACS buffer followed by fixation and permeabilisation using the Foxp3/Transcription Factor Staining Buffer Set (eBioscience, San Diego, California, USA) according to the manufacturer’s instructions. Briefly, cells were stained with rat anti-mouse Foxp3 Alexa Fluor 700 (clone FJK-16, eBioscience, San Diego, California, USA) in permeabilisation buffer, washed twice in the same buffer and resuspended in FACS buffer. Samples were then analysed with a BD LSR Fortessa (BDBiosciences, University of Dundee, Central Services) followed by FlowJo version 7.6.4.

### Renal histology and assessment of disease severity

The right kidney was used for assessing lupus nephritis severity by light microscopy. Kidneys were fixed in 10% neutral-buffered formalin. Processing and staining of kidney sections was conducted by the Veterinary Services, University of Glasgow, UK. Kidney sections (5 μm) were stained with H&E or Masson’s trichrome and evaluated by light microscopy. Scoring of nephritis severity included examining glomeruli, tubular and perivascular regions, as described previously [20]. Glomerular pathology was assessed by counting the number of cells per glomerular cross-section (gcs), of 30 glomeruli per kidney. The scoring system was as follows; score 0, normal cellularity (35 to 40 cells/gcs); 1, mild hypercellularity (41 to 50 cells/gcs); 2, moderate hypercellularity (51 to 60 cells/gcs) with segmental or diffuse proliferative changes; 3, severe hypercellularity (>60 cells/gcs) with segmental or global sclerosis, crescent formation and severe exudation. Tubular pathology was assessed based on the percentage of tubules showing dilatation or atrophy among 200 tubules in a randomly chosen field. Perivascular cell accumulation was also evaluated by counting the number of cell layers surrounding randomly chosen inter- and intralobular arteries. Perivascular scoring included; 0, no perivascular infiltrate; 1, less than 5 layers surrounding less than half the diameter of the artery; 2, 5 to 10 layers surrounding more than half of the diameter of the vessel; 3, more than 10 layers surrounding more than half the diameter of the artery). Scoring of all kidney sections was done in a blinded fashion.

### Immunohistochemistry

Kidneys were snap-frozen and stored at −80°C until sectioned: 5-μM cryostat sections were prepared, air-dried and fixed in acetone for 10 minutes. Rat anti-mouse monoclonal antibodies against Sn (clone SER4 and 3D6, in-house), CD68 (clone FA-11, eBioscience, San Diego, California, USA), CD4 (clone L3T4, eBioscience, San Diego, California, USA) and Foxp3 (clone FJK-16, eBioscience, San Diego, California, USA) were used to assess the presence of different cell populations. HRP-labelled goat anti-rat Ig was used as secondary antibody (mouse-adsorbed; Vector laboratories, Peterborough, UK). Spleen tissues from Sn wild-type and deficient mice were used as positive and negative controls for Sn, respectively.

### Statistical analysis

Statistical significance was determined by the Mann–Whitney test. A *P*-value was considered significant when less than 0.05.

## Results

### Sn expression is upregulated in renal tissue of diseased NZBWF1 mice

**Figure 1:**
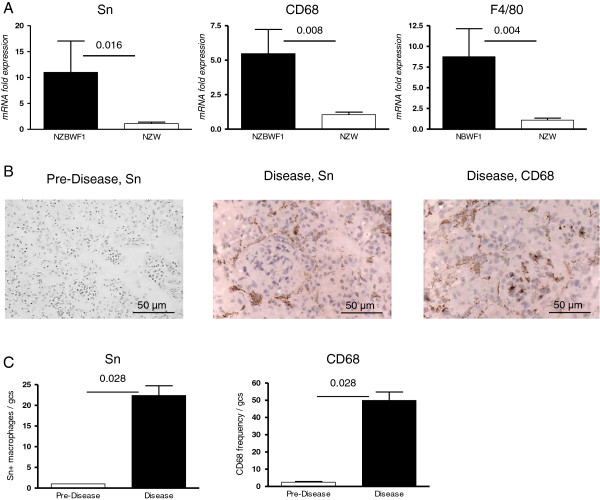
**Upregulation of renal sialoadhesin (Sn) expression following disease onset in BWF1 mice. (A)** Renal cortical tissues from 28-week proteinuric BWF1 mice and age -atched NZW mice were examined for gene expression of Sn, CD68 and F4/80 (n = 4 per group) by qRT-PCR. **(B)** Immunohistochemistry of frozen kidney sections from pre-disease (10 weeks) and diseased (28 weeks) NZBWF1 mice, indicating the anatomical locations of CD68^+^ and Sn^+^ macrophages in renal tissues of diseased NZBWF1 mice. **(C)** Quantification of Sn^+^ macrophages in glomerular cross-sections (gcs). Statistical analysis was done using the Mann–Whitney test and *P*-values are indicated.

Recent analyses of Sn^+^ monocytes in patients with active SLE showed an increase in their frequency that matched disease activity [12,13]. However, it is unclear whether Sn expression is upregulated on macrophages in diseased renal tissues. To this end, we examined the expression of Sn and pan-macrophage lineage markers CD68 and F4/80 in renal tissues of 28 week old proteinuric NZBWF1, age matched NZW and pre-disease NZBWF1 mice by qRT-PCR. This revealed that dramatic upregulation in the mRNA expression of Sn, CD68 and F4/80 occurs in proteinuric NZBWF1 mice (Figure 1A). There were no significant differences between pre-disease NZBWF1 and control NZW mice (data not shown). In order to confirm the expression of Sn at a protein level, we used immunohistochemical staining for Sn and CD68 in renal tissues from NZBWF1 mice. Consistent with the qRT-PCR analysis, Sn was absent in pre-diseased kidneys and increased in renal tissues of diseased NZBWF1 mice (Figure 1B and C). Interestingly, Sn^+^ macrophages assumed a distinct periglomerular and tubulointerstitial distribution and, in contrast to CD68^+^ macrophages, were not found within the glomerular capillaries (Figure 1B).

**Figure 2:**
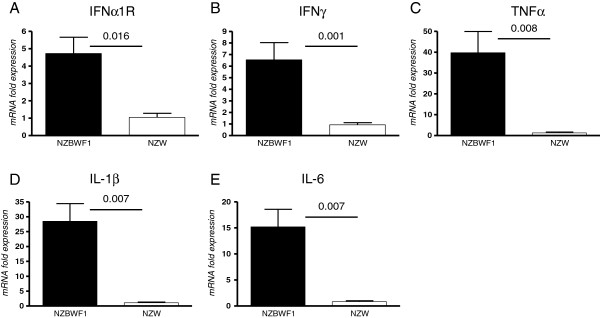
**Upregulation of inflammatory markers in renal tissues from diseased New Zealand black x New Zealand white F1 (NZBWF1) mice. (A**-**E)** Gene expression in kidneys of NZBWF1 mice relative to New Zealand white (NZW) control tissues was determined for the indicated markers by qRT-PCR using five mice per group. Statistical analysis was done using the Mann–Whitney test and *P*-values are indicated.

Concomitant with upregulation of Sn in diseased renal tissues of NZBWF1 mice, we also observed increased expression of the *Ifnar1* gene (Figure 2A), similar to what has been reported previously in lupus-prone MRL/lpr mice as part of the interferon signature [21]. In addition, genes encoding several pro-inflammatory cytokines were found to be upregulated in their expression, including IFNγ, TNFα, IL-1β and IL-6 (Figure 2). Taken together, these data show that Sn is upregulated in kidney tissues from nephritic NZBWF1 mice and this is associated with enhanced expression of a number of inflammatory cytokines.

### Sialoadhesin deficiency does not affect the severity of lupus nephritis in NZBWF1 mice

**Figure 3:**
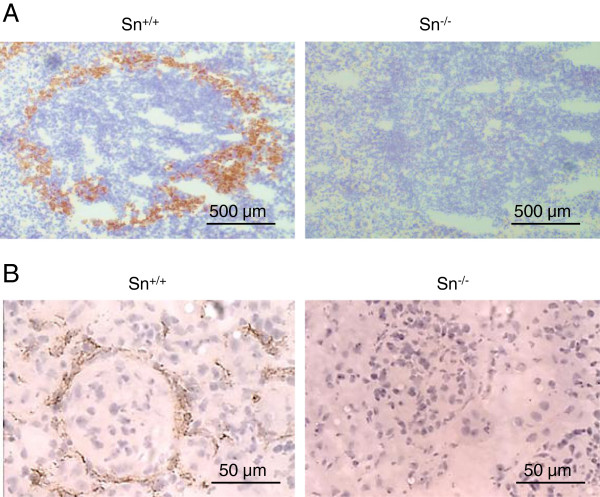
**Generation of sialoadhesin (Sn)-deficient NZBWF1 mice.** Frozen sections from spleen **(A)** and kidney **(B)** of Sn^+/+^ and Sn^−/−^ NZBWF1 mice were stained for Sn using immunohistochemistry.

**Figure 4:**
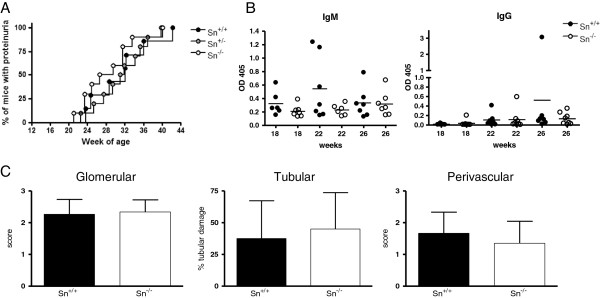
**Disease severity in sialoadhesin (Sn)**^**+/+ **^**and Sn**^**−/− **^**NZBWF1 mice. (A)** Disease onset and progression was determined by measurement of proteinuria in each of the indicated genotypes; n = 7, 10 and 10 mice per group for Sn^+/+^, Sn^+/−^ and Sn^−/−^ respectively. **(B)** Serum immunoassays of IgM and IgG anti-dsDNA at 18, 22 and 26 weeks of age. **(C)** Light microscopy-based scoring of disease severity in glomerular, tubulointerstitial and perivascular compartments in 28-week old mice; n = 6 mice per group.

To investigate whether the strong upregulation of Sn is important in the pathogenesis of lupus nephritis, we generated Sn-deficient NZBWF1 mice as described in Methods. Sn deficiency was confirmed by means of PCR (data not shown) and immunohistochemistry of spleen and kidney sections (Figure 3). Age-matched Sn^+/+^, Sn^+/−^ and Sn^−/−^ mice were monitored for disease onset (proteinuria) and progression leading to death (Figure 4A). No significant differences were observed. However, 3 Sn^−/−^ mice showed early proteinuria at weeks 16, 17 and 21, which subsided and then reappeared at weeks 21, 25 and 31 respectively, progressing to death. A fourth Sn-deficient mouse developed early proteinuria at week 21 which progressed. The earliest onset of proteinuria in Sn-expressing mice was at week 23 and in all cases, disease was progressive (Figure 4A). This difference in the early onset of proteinuria between Sn-deficient mice and Sn-expressing mice was significant (Fishers exact test, *P* = 0.04).

The analysis of serial serum samples from Sn^+/+^ and Sn^−/−^ NZBF1 mice for IgM and IgG anti-dsDNA showed no significant differences between the two groups (Figure 4B). The severity of lupus nephritis was also assessed histologically using the World Health Organization (WHO) scoring system as outlined in Methods. There were no significant differences in glomerular, tubulointerstitial or perivascular scores between Sn^−/−^ and Sn^+/+^ mice (Figure 4C). Taken together, these data demonstrated that Sn deficiency does not significantly influence the progression to end-stage nephritis in NZBWF1 mice.

### Sn^−/−^ NZBWF1 mice have higher frequency of activated CD4^+^ T cells

**Figure 5:**
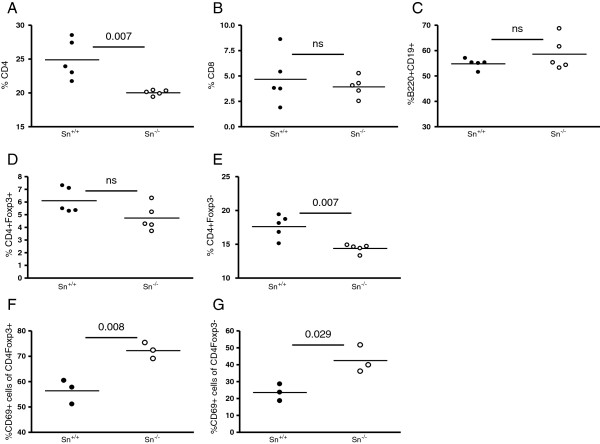
**Flow cytometry of splenocytes from diseased sialoadhesin (Sn)+/+ and Sn−/− New Zealand black x New Zealand white F1 (NZBWF1) mice. (A**-**G)** Splenocytes were isolated from 28-week-old diseased Sn+/+ (filled circles) and Sn−/− (open circles) NZBWF1 mice. Each circle represents an individual mouse. Statistical analysis was done using the Mann–Whitney test and *P*-values are indicated; ns, not significant.

We have previously shown that Sn can interact with Sn ligands (SnL) induced on a subset of highly activated, potentially pathogenic CD4^+^Foxp3^-^ effector T cells (Teffs) and trigger their apoptosis [22]. SnL are also upregulated on subsets of CD4^+^FoxP3^+^ regulatory T cells (Tregs) in experimental autoimmune encephalomyelitis (EAE), leading to an Sn-dependent reduction in numbers of Tregs and exacerbation of disease [17]. In order to examine the impact of Sn deficiency on different splenocyte subsets, we performed flow cytometric analysis (Figure 5). This revealed a significant reduction in the percentage of CD4^+^ T cells in Sn^−/−^ mice (Figure 5A), but there was no difference in CD8^+^ or CD19^+^B220^+^ cells (Figure 5B and C). This reduction was observed in both Foxp3^+^ and Foxp3^-^ CD4^+^ subsets (Figure 5D and E). However, the percentages of CD4^+^Foxp3^+^ and CD4^+^Foxp3^-^ T cells that expressed the activation marker CD69 were both significantly increased in spleens of Sn^−/−^ mice (Figure 5F and G). As activated CD4^+^ T cells express SnL [22], this is consistent with a negative regulatory role of Sn on both Teffs and Tregs in SLE.

**Figure 6:**
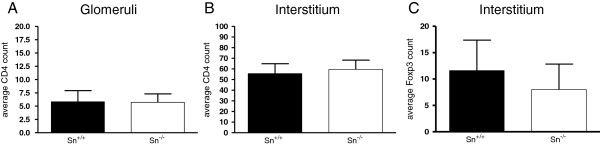
**The frequency of CD4+ and Foxp3+ T cells in glomerular and tubulointerstitial compartments of sialoadhesin (Sn)+/+ and Sn−/− New Zealand black x New Zealand white F1 (NZBWF1) mice. (A**-**C)** Immunohistochemistry staining for CD4 and Foxp3 was done on renal sections from five mice per group. No significant differences were observed using the Mann–Whitney test.

In order to analyse the effect of Sn deficiency on CD4^+^ T cells in renal tissues of NZBWF1 mice, we examined the frequency of CD4^+^ and Foxp3^+^ T cells using immunohistochemistry. There were no differences in the frequency of CD4^+^ T cells in glomeruli and tubulointerstitial tissues between Sn^+/+^ and Sn^−/−^ mice (Figure 6A and B). We were unable to demonstrate positive Foxp3 staining in the glomerular compartment, but the tubulointerstitial tissues showed similar numbers of Foxp3^+^ cells in Sn^+/+^ and Sn^−/−^ mice (Figure 6C).

## Discussion

The expression and function of Sn have been investigated in a number of inflammatory conditions in animal models and in humans [10,13-17,23-29]. The majority of these studies suggested a pro-inflammatory role for the molecule. Indeed, Sn deficiency in mice has been reported to be associated with disease amelioration in several autoimmune conditions [14-17]. Although increased Sn levels were found to be representative of disease activity in human SLE [12], it has remained unclear whether Sn plays a direct role in the disease pathogenesis. In this study, we found abundant Sn expression at the mRNA and protein levels in the kidneys of nephritic mice. In addition, a number of cytokines that have been implicated previously in the induction of Sn under inflammatory conditions were also upregulated [30]. As there was a parallel increase in gene expression of the pan macrophage markers CD68 and F4/80 in the nephritic mice, it is difficult to differentiate between an enhanced induction of Sn on renal macrophage subsets and generalised infiltration of kidney tissues by macrophages. However, immunohistochemistry analysis revealed that Sn^+^ macrophages were mainly distributed in a periglomerular manner and absent from the glomerular tuft, unlike CD68^+^ macrophages. The significance of this preferential anatomical location of Sn^+^ macrophages is currently unclear.

Previously, Sn expression on macrophages in a model of EAE was reported to downregulate numbers of SnL^+^CD4^+^Foxp3^+^ Tregs via Sn-SnL interactions, leading to increased disease severity [17]. Recently, we confirmed the abundance of SnL on Tregs and also identified a novel subset of activated CD4^+^Foxp3^-^ Teffs bearing SnL following TCR ligation *in vitro*[22]. A phenotypically similar subset of SnL^+^CD4^+^Foxp3^-^ Teffs was identified in proteinuric NZBWF1 mice, with the frequency of this subset correlating closely with disease status (proteinuria presence versus absence) [22]. We therefore hypothesized that Sn deficiency might be associated with an augmented disease severity due to an increase in these highly activated CD4^+^Foxp3^-^ T cells. Surprisingly, however, despite an earlier onset of proteinuria, the disease progression was not different between Sn^+/+^ and Sn^−/−^ mice. Since the frequency of both activated Teffs and Tregs was enhanced in the spleens of Sn^−/−^ mice, the net outcome on CD4 T cell function could be neutral and help explain why the severity of nephritis was similar regardless of the genotype. Importantly, our results indicate that unlike non-polygenic autoimmune conditions studied previously [14-17], Sn deficiency does not affect disease severity in SLE.

In conclusion, our findings suggest that Sn expression is increased in murine lupus nephritis and correlates with histological severity. However, Sn deficiency does not affect the disease progression or histological severity. Collectively, these data indicate that the role of Sn in SLE is redundant. As demonstrated by others [12,13], Sn serves as a potentially useful biomarker of inflammation and disease severity in SLE.

## Conclusions

In the NZBWF1 model of murine lupus nephritis, Sn expression in the kidney was increased and correlated with histological severity of disease. Although Sn deficiency was associated with dynamic changes of splenic activated CD4 T cells and earlier onset of disease, it did not affect disease progression or histological severity. Collectively, these data suggest that Sn acts as a biomarker that is reflective of disease in murine lupus nephritis rather than playing a causal role.

## Abbreviations

BSA: Bovine serum albumin; EAE: Experimental autoimmune encephalomyelitis; EDTA: Ethylenediaminetetraacetic acid; ELISA: Enzyme-linked immunosorbent assay; FACS: Fluorescence-activated cell sorting; gcs: Glomerular cross-section; H&E: Haematoxylin and eosin; HRP: Horse radish peroxidase; IFN: Interferon; Ig: Immunoglobulin; NZB: New Zealand black; NZBWF1: New Zealand black x New Zealand white F1; NZW: New Zealand white; PBS: Phosphate-buffered saline; PBST: Phosphate-buffered saline plus 0.05% Tween; qRT-PCR: Quantitative reverse transcriptase-polymerase chain reaction; SLE: Systemic lupus erythematosus; Sn: Sialoadhesin; SnL: Sn ligands; Teffs: CD4 + Foxp3-effector T cells; TNF: Tumour necrosis factor; Tregs: CD4 + Foxp3+ regulatory T cells; WT: Wild-type.

## Competing interests

The authors declare that they have no competing interests.

## Supplementary Material

Additional file 1 **NZW and NZB susceptibility loci and corresponding markers used in generating BWF1 mice deficient for sialoadhesin (Sn).** Description: Table showing New Zealand white (NZW) and New Zealand black (NZB) susceptibility loci and corresponding markers used in generating BWF1 mice deficient for Sn. Click here for file
