# Supplementary material for: Sialoadhesin deficiency does not influence the severity of lupus nephritis in New Zealand Black x New Zealand White F1 mice
Source: Arthritis Res Ther. 2013 Nov 1;15(6):R175. doi: 10.1186/ar4364 (PMC3978688; doi:10.1186/ar4364)
Supplement: Additional file 1 — NZW and NZB susceptibility loci and corresponding markers used in generating BWF1 mice deficient for sialoadhesin (Sn). Description: Table showing New Zealand white (NZW) and New Zealand black (NZB) susceptibility loci and corresponding markers used in generating BWF1 mice deficient for Sn. [file ar4364-S1.docx]

**Additional file 1: Table S1.** NZW and NZB susceptibility loci and corresponding markers used in generating BWF1 mice deficient for Sn.

| NZW loci and markers | | |  | NZB loci and markers | | |
| --- | --- | --- | --- | --- | --- | --- |
| Locus | Chromosome | Marker |  | Locus | Chromosome | Marker |
| Sle1 | 1 | D1Mit1001 |  | Sle1 | 1 | D1Mit132 |
| Sle1 | 1 | D1Mit111 |  | Sle1 | 1 | D1Mit308 |
| Sle1 | 1 | D1Mit155 |  | Sle1 | 1 | D1Mit111 |
| Wbw1 | 2 | D2Mit66 |  | Sle2 | 4 | D4Mit193 |
| Wbw1 | 2 | D2Mit411 |  | Sle2 | 4 | D4Mit17 |
| Wbw1 | 2 | D2Mit148 |  | Sle2 | 4 | D4Mit308 |
| Nbwa2 | 4 | D4Mit193 |  | Lbw4 | 6 | D6Mit209 |
| Nbwa2 | 4 | D4Mit308 |  | Lbw4 | 6 | D6Mit36 |
| Lbw3 | 5 | D5Mit95 |  | Lbw4 | 6 | D6Mit328 |
| Lxw2 | 6 | D6Mit138 |  | Sle3 | 7 | D7Mit350 |
| Lxw2 | 6 | D6Mit36 |  | Sle3 | 7 | D7Mit101 |
| Lbw5 | 7 | D7Mit294 |  | Lmb4 | 10 | D10Mit230 |
| Lbw5 | 7 | D7Mit350 |  | Lmb4 | 10 | D10Mit96 |
| Nbwa1 | 12 | D12Mit182 |  | Lbw8 | 11 | D11Mit285 |
| Nbwa1 | 12 | D12Mit158 |  | Lbw8 | 11 | D11Mit333 |
| Nw | 13 | D13Mit275 |  | B6.NZBc13 | 13 | D13Mit16 |
| Nw | 13 | D13Mit260 |  | B6.NZBc13 | 13 | D13Mit74 |
| Nwa1 | 16 | D16Mit131 |  | H2 | 17 | D17Mit51 |
| Nwa1 | 16 | D16Mit52 |  | H2 | 17 | D17Mit10 |
| Lbw1 | 17 | D17Mit245 |  | Fas | 19 | D19Mit333 |
| Wbw2 | 17 | D17Mit93 |  |  | | |
| Lbw6 | 18 | D18Mit177 |  |  |  |  |
| Lbw6 | 18 | D18Mit48 |  |  |  |  |
| Nwa2 | 19 | D19Mit28 |  |  |  |  |
| Nwa2 | 19 | D19Mit33 |  |  |  |  |
